# Effect of Korean medicine treatment on surgery and opioid prescription among patients with lumbar spinal stenosis: a nationwide retrospective cohort study

**DOI:** 10.3389/fmed.2026.1703911

**Published:** 2026-01-16

**Authors:** Won-Jeong Ha, Ho-Yeon Go, In-Hyuk Ha, Yoon Jae Lee

**Affiliations:** 1 Jaseng Hospital of Korean Medicine, Seoul, Republic of Korea; 2 Department of Korean Internal Medicine, Semyung University, Jecheon-si, Chungcheongbuk-do, Republic of Korea; 3 Jaseng Spine and Joint Research Institute, Jaseng Medical Foundation, Seoul, Republic of Korea

**Keywords:** lumbar spinal stenosis, Korean medicine, opioid, surgery, real-world data, real-world evidence

## Abstract

**Introduction:**

Lumbar spinal stenosis (LSS) is a prevalent degenerative spinal condition in older adults, often necessitating surgical intervention and long-term pharmacological treatment. Korean medicine (KM) has emerged as a relatively safe alternative; however, its impact on surgical rates and opioid use in patients with LSS has not been thoroughly investigated. This nationwide retrospective cohort study aimed to assess the effects of KM treatment on these outcomes.

**Methods:**

We analyzed claims data from the Health Insurance Review and Assessment Service (HIRA) for patients newly diagnosed with LSS in 2015. The KM group included patients who had ≥ 3 outpatient KM visits within 1 year of diagnosis and received KM care more frequently than Western medicine (WM). The control group comprised patients with ≥ 3 outpatient WM visits and no KM use during the same period. Propensity score matching (PSM) was performed, and outcomes were compared using Kaplan–Meier survival analysis and Cox proportional hazards models.

**Results:**

After PSM, 70,897 matched pairs were included in the surgery dataset, and 17,217 patients per group in the opioid dataset. KM treatment was associated with significantly lower risks of lumbar surgery (hazard ratio [HR]: 0.821; 95% confidence interval [CI]: 0.782–0.862), opioid use (HR: 0.810; 95% CI: 0.752–0.872), and opioid use excluding tramadol (HR: 0.76; 95% CI: 0.630–0.919).

**Conclusion:**

These findings suggest that KM treatment is associated with a reduced long-term risk of lumbar surgery and opioid use in patients with LSS. KM may represent a potentially effective conservative treatment option. Further randomized controlled trials are warranted to validate these findings.

## 1 Introduction

Lumbar spinal stenosis (LSS) is a condition characterized by the progressive compression of neural and vascular structures within the spinal canal, resulting from degenerative hypertrophy of bones, ligaments, and synovial tissues in the lower axial spine. This narrowing can lead to a range of symptoms, including lower back pain, radiating leg pain, and neurogenic claudication, either individually or in combination (1).

LSS affects approximately 103 million individuals worldwide each year (2). It is one of the most common degenerative spinal disorders and is strongly associated with aging. Notably, 47% of individuals aged 60 to 69 have been reported to have mild to moderate stenosis (3), and LSS is the leading indication for spinal surgery among adults aged 65 years and older (4).

With the ongoing global increase in the elderly population, both the prevalence of LSS and its associated healthcare and societal burdens are expected to rise. Moreover, LSS has been shown to have a more profound negative impact on quality of life (QoL) than other comorbid conditions, such as hip or knee osteoarthritis and cardiovascular disease (5), highlighting the urgent need for more effective treatment strategies.

The treatment of LSS typically follows a stepwise approach. Conservative management, including physical therapy and pharmacological interventions, is usually initiated first. However, the long-term effectiveness of these conservative strategies remains uncertain. Physical therapy often provides only temporary symptom relief (6), and epidural steroid injections (ESIs) provide short-term symptom relief but show limited long-term effectiveness, as a systematic review and meta-analysis of randomized controlled trials reported only a modest, non-significant short-term trend toward reduced surgery rates and no sustained benefit over longer follow-up (7). When symptoms persist or worsen despite conservative measures, surgical decompression becomes an option.

However, surgery carries considerable risk and limited long-term success, particularly among older adults. A prospective study reported that 33% of patients who underwent surgery experienced treatment failure (8), and a retrospective cohort study using data from the Korean National Health Insurance Service showed that the reoperation rate after surgery for LSS reached 14.2% within 5 years and was projected to increase to 22.9% by year 10 (9). Furthermore, among older adults with musculoskeletal pain, the use of opioid analgesics has been linked to a threefold higher risk of adverse events compared with placebo, and the likelihood of treatment discontinuation due to adverse effects is four times higher (10). These limitations highlight the need for safe and sustainable treatment alternatives.

Korean medicine (KM) has gained attention as a safe alternative. In clinical practice in Korea, various KM modalities – including acupuncture, herbal medicine, pharmacopuncture, and Chuna manual therapy – are frequently used to manage LSS. Several studies have demonstrated that these KM interventions can improve pain, physical function, and QoL (11–15). Acupuncture and pharmacopuncture are known to modulate inflammatory pathways, enhance microcirculation, and regulate central pain signaling through both peripheral and central mechanisms (16). Chuna therapy may relieve mechanical compression and improve spinal alignment (17). These multimodal mechanisms may collectively alleviate pain, improve function, and potentially reduce the need for invasive surgery or long-term opioid use.

However, to date, no study has evaluated the effectiveness of KM using large-scale, population-based data from real-world clinical settings in Korea. Previous studies were generally limited in scope and duration, making it difficult to clarify the long-term, population-level impact of KM on major clinical outcomes. In particular, the effect of KM treatment on surgical rates and opioid use among patients with LSS has not been examined using representative national data. Addressing these evidence gaps, the present study aimed to evaluate the real-world effectiveness of integrative KM treatment for LSS, focusing specifically on its association with lumbar surgery and opioid use.

## 2 Materials and methods

### 2.1 Data source

This study utilized a nationwide claims database provided by the Health Insurance Review and Assessment Service (HIRA) of Korea (18), comprising healthcare claims for services reimbursed between January 1, 2014, and December 31, 2020.

The study received ethical exemption approval from the Institutional Review Board of Jaseng Hospital of Korean Medicine (JASENG IRB 2021-07-002; approved on July 9, 2021). As the research involved secondary analysis of de-identified, publicly available data, the requirement for informed consent was waived. All analyses were conducted in accordance with the principles outlined in the Declaration of Helsinki. The study protocol was reviewed and approved by HIRA for the use of public data, and all procedures adhered to relevant ethical and regulatory guidelines.

### 2.2 Study population

Patients were eligible for inclusion if they received healthcare services with LSS (ICD-10 code M48.0) listed as the primary diagnosis between January 1 and December 31, 2015. Medical service utilization was defined as any claim submitted under the National Health Insurance (NHI) system for LSS-related treatment, including KM interventions such as acupuncture, electroacupuncture, and cupping.

**Figure 1:**
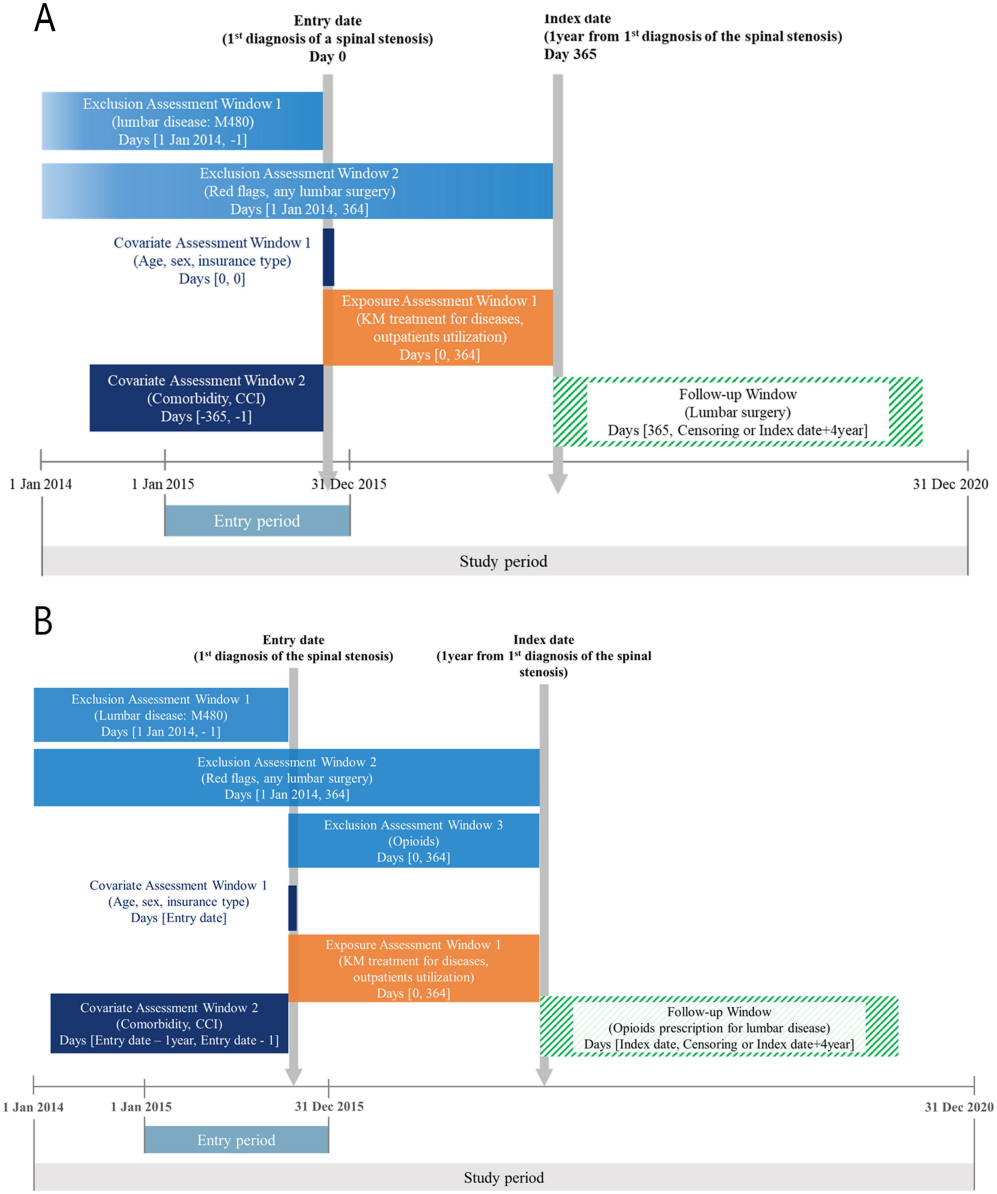
Overview of the study design. **(A)** Surgery set; **(B)** Opioid set.

The entry date was defined as the first diagnosis of LSS (M48.0) in 2015, while the index date was 1 year after the entry date. A one-year washout period prior to the entry date was applied to exclude patients with a prior diagnosis of LSS. Patients with red flag conditions (e.g., malignancy, infection, or major trauma) diagnosed within 1 year before the index date were excluded (19) (see Supplementary Table 1). Patients with a history of spinal surgery, other than surgeries related to study outcomes, were also excluded. For the opioid analysis, individuals who had been prescribed opioid analgesics within 1 year before the entry date were excluded (Figure 1).

The Charlson Comorbidity Index (CCI) was calculated based on ICD-10 codes using the method proposed by Quan et al. (20). Medical service utilization was quantified by counting LSS-related visits (to either KM or Western medicine [WM] providers) between the entry and index dates, categorized into quartiles, and used as a proxy variable for disease severity.

### 2.3 Definition of KM and non-KM groups

Customized claims data extracted from HIRA based on variables requested by the researchers were utilized. Patients diagnosed with LSS (M48.0) were categorized into two groups based on their healthcare utilization patterns within the first year after diagnosis:

(a) The KM group included patients who had ≥3 outpatient visits to KM providers and had more KM visits than WM visits during the same period. (b) The non-KM group comprised patients who had ≥3 outpatient WM visits and no KM visits during the one-year period following the entry date. To evaluate the robustness of the operational definition of KM utilization, we additionally conducted sensitivity analyses applying higher visit thresholds (≥6 and ≥8 KM visits within the first year).

#### 2.3.1 Outcome measures

The primary outcomes were: (a) lumbar spine surgery, defined as any of the following procedures: discectomy, laminectomy, or spinal fusion. Relevant procedure codes from the NHI claims database are listed in Supplementary Table 2 (21). (b) Opioid prescription, defined as either ≥14 days of tramadol-based analgesics or ≥7 days of opioid-class analgesics (22). Analyses were conducted for (a) any qualifying opioid use (including tramadol), and (b) opioid-only use (excluding tramadol). Medications were categorized using Anatomical Therapeutic Chemical (ATC) codes (see Supplementary Table 3). The 7-day threshold for opioid-class analgesics was based on clinical guidelines indicating that prolonged opioid use beyond 3–7 days in acute pain offers no additional benefit and may be associated with poorer functional outcomes (23). The 14-day threshold for tramadol was determined according to opioid prescribing guidelines (24) and previous observational research that defined exposure windows using clinically interpretable and empirically derived criteria (25).

The follow-up period began 1 year after the entry date and extended up to 4 years post-entry. Patients were censored from the analysis if they, prior to the outcome event, (1) were prescribed opioids for unrelated conditions, (2) were diagnosed with red flag conditions, or (3) underwent lumbar surgery not meeting the outcome criteria.

### 2.4 Statistical analysis

Descriptive statistics were used to summarize patient demographics, LSS-related healthcare utilization, comorbidities, and CCI scores. Categorical variables were reported as frequencies (*n*) and percentages (%), while continuous variables were expressed as means and standard deviations (SD). Baseline differences between KM and non-KM groups were assessed using the chi-square or Fisher’s exact test for categorical variables, and the independent *t*-test for continuous variables.

To minimize selection bias and control for confounding factors affecting surgery or opioid outcomes, propensity score matching (PSM) was applied. Propensity scores were calculated using logistic regression, and 1:1 nearest-neighbor matching was performed with a caliper width of 0.1. Matching variables included: sex, age, CCI, presence of spondylolisthesis, and hospitalization status and insurance type, which also served as a proxy for socioeconomic status. Standardized mean differences (SMDs) were used to assess covariate balance post-matching, with an SMD > 0.1 indicating imbalance.

Kaplan–Meier survival curves and log-rank tests were used to compare time-to-event outcomes between groups. Cox proportional hazards regression models were employed to estimate hazard ratios (HRs) and 95% confidence intervals (CIs). The Akaike Information Criterion (AIC) was used to evaluate model fit across different stages of adjustment and PSM.

All analyses were performed using SAS Enterprise Guide version 7.1 (SAS Institute Inc., Cary, NC, United States). A two-sided *p*-value of <0.05 was considered statistically significant.

## 3 Results

### 3.1 Demographic characteristics and propensity score matching

A total of 1,229,897 patients diagnosed with LSS (ICD-10 code M48.0) between January 1 and December 31, 2015, were initially identified. Patients were excluded if they (1) had a diagnosis of LSS within 1 year prior to the entry date, (2) were diagnosed with red flag conditions within 1 year before the index date, or (3) underwent lumbar surgery before the index date. After applying these criteria, 559,121 patients were included in the final study cohort.

**Figure 2:**
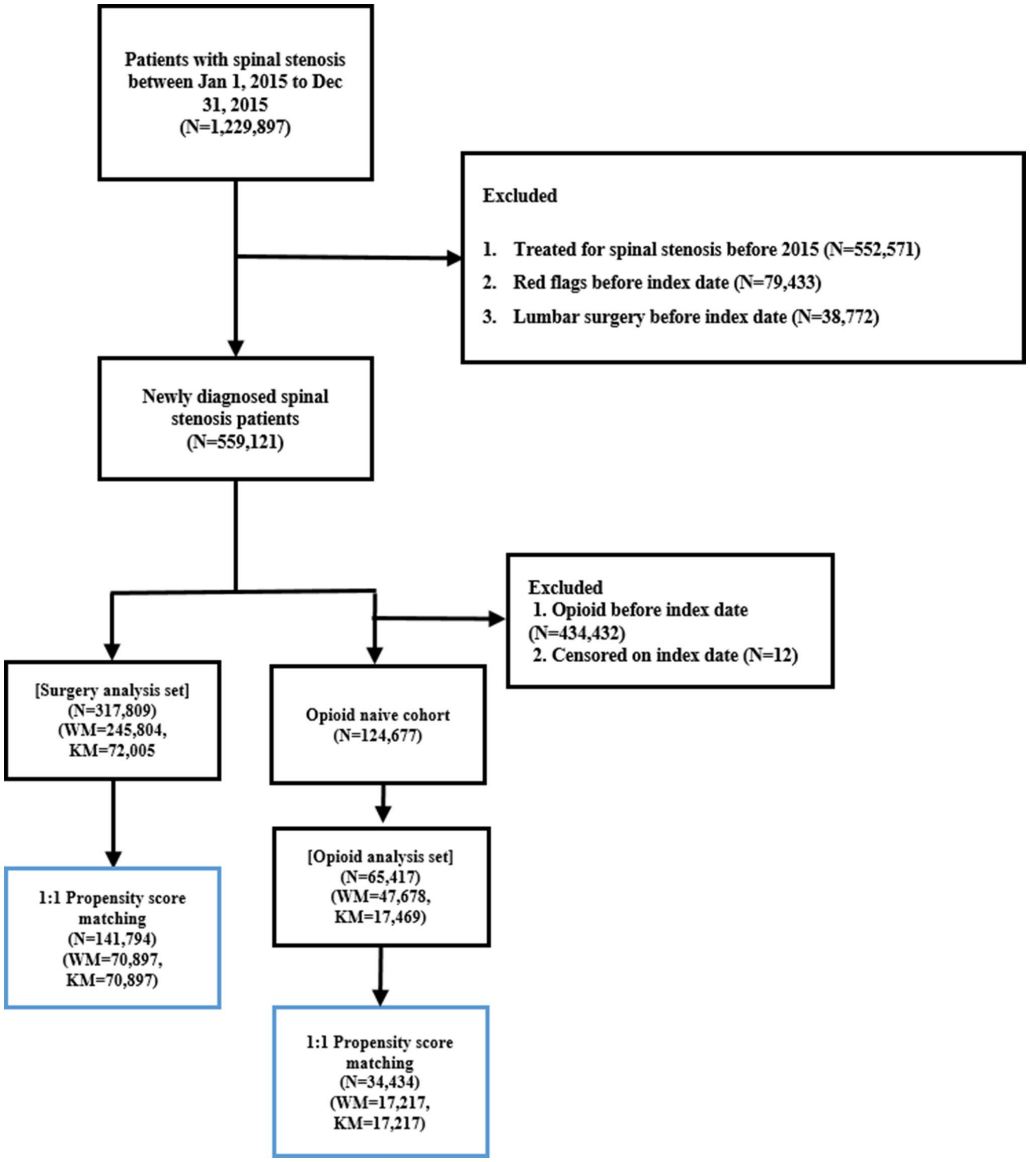
Flowchart illustrating the inclusion and exclusion criteria applied to identify the final study cohorts for the surgery and opioid analyses.

Patients were classified into two groups based on healthcare utilization patterns during the one-year period following diagnosis. The KM group included those who had ≥ 3 outpatient visits to KM institutions and had more KM visits than WM visits during that year. The control group comprised patients who had ≥ 3 outpatient WM visits and no KM visits in the same period. Based on these criteria, the surgery dataset included 317,809 patients, with 72,005 in the KM group and 245,804 in the control group, while the opioid dataset comprised 65,417 patients, including 47,678 in the KM group and 17,469 in the control group (Figure 2).

Prior to PSM, significant baseline differences were observed between the KM and control groups in both datasets. These differences included sex, age, type of insurance coverage, CCI, hospitalization status, and the presence of spondylolisthesis. The KM group had a higher proportion of females and older patients, as well as a greater number of patients covered by the NHI. Additionally, the KM group tended to have higher CCI scores and a higher prevalence of spondylolisthesis. Similar patterns were observed in the opioid dataset.

To address these differences, PSM was applied. After matching, the surgery dataset included 70,897 matched patients in each group, while the opioid dataset included 17,217 matched patients in each group. Following PSM, no statistically significant differences in baseline characteristics were observed between the KM and control groups. All SMDs were 0.00, indicating excellent covariate balance (Table 1). Additional comparisons of comorbidities and healthcare utilization not included in the PSM model are presented in Supplementary Table 4.

**Table 1:** Baseline characteristics of the study population.

Variables	Before propensity score matching						After propensity score matching					
	WM		KM		*p*-value	SMD	WM		KM		*p*-value	SMD
	*N*	%	*N*	%			*N*	%	*N*	%		
Surgery dataset	245,804		72,005				70,897		70,897			
Sex												
Male	93,496	38.0	22,042	30.6	<0.001	0.16	21,713	30.6	21,712	30.6	0.317	0.00
Female	152,308	62.0	49,963	69.4		−0.16	49,184	69.4	49,185	69.4		0.00
Age group												
<50	24,017	9.8	6,273	8.7	<0.001	0.04	6,037	8.5	6,038	8.5	1.000	0.00
50–59	55,162	22.4	14,029	19.5		0.07	13,644	19.2	13,644	19.2		0.00
60–69	74,380	30.3	22,284	31.0		−0.01	22,009	31.0	22,009	31.0		0.00
70–79	69,299	28.2	23,609	32.8		−0.10	23,445	33.1	23,444	33.1		0.00
≥80	22,946	9.8	5,810	8.1		0.05	5,762	8.1	5,762	8.1		0.00
Insurance type												
NHI	226,806	92.3	68,400	95.0	<0.001	−0.11	67,348	95.0	67,348	95.0	1.000	0.00
Medical aid	18,998	7.7	3,605	5.0		0.11	3,549	5.0	3,549	5.0		0.00
CCI												
0	132,580	53.9	38,105	52.9	<0.001	0.02	37,417	52.8	37,417	52.8	0.920	0.00
1	72,167	29.4	21,511	29.9		−0.01	21,223	29.9	21,222	29.9		0.00
2	26,061	10.6	7,929	11.0		−0.01	7,850	11.1	7,849	11.1		0.00
≥3	14,996	6.1	4,460	6.2		0.00	4,407	6.2	4,409	6.2		0.00
WM hospitalization												
No	222,884	90.7	65,578	91.1	0.001	−0.01	65,359	92.2	65,358	92.2	0.317	0.00
Yes	22,920	9.3	6,427	8.9		0.01	5,538	7.8	5,539	7.8		0.00
KM hospitalization												
No	245,804	100	70,897	98.5	<0.001	0.18	70,897	100	70,897	100	–	–
Yes			1,108	1.5		−0.18						
Spondylolisthesis												
No	237,491	96.6	69,374	96.4	<0.001	0.01	38,352	96.4	68,351	96.4	0.317	0.00
Yes	8,313	3.4	2,631	3.6		−0.01	2,545	3.6	2,545	3.6		0.00
Opioid dataset	47,678		17,469				17,217		17,217			
Sex												
Male	19,628	41.2	5,851	33.5	<0.001	0.16	5,762	33.5	5,764	33.5	0.157	0.00
Female	28,050	58.8	11,618	66.5		−0.16	11,455	66.5	11,453	66.5		0.00
Age group												
<50	6,333	13.3	2,028	11.6	<0.001	0.05	1,964	11.4	1,964	11.4	1.000	0.00
50–59	11,252	23.6	3,662	21.0		0.06	3,571	20.7	3,571	20.7		0.00
60–69	14,135	29.7	5,389	30.9		−0.03	5,332	31.0	5,332	31.0		0.00
70–79	11,965	25.1	5,066	29.0		−0.09	5,035	29.2	5,035	29.2		0.00
≥80	3,993	8.4	1,324	7.6		0.03	1,315	7.6	1,315	7.6		0.00
Insurance type												
NHI	45,071	94.5	16,905	96.8	<0.001	−0.11	16,662	96.8	16,662	96.8	1.000	0.00
Medical aid	2,607	5.5	564	3.2		0.11	555	3.2	555	3.2		0.00
CCI												
0	29,187	61.2	10,422	59.7	0.004	0.03	10,250	59.5	10,250	59.5	0.766	0.00
1	12,690	26.6	4,803	27.5		−0.02	4,749	27.6	4,748	27.6		0.00
2	3,869	8.1	1,504	8.6		−0.02	1,492	8.7	1,489	8.7		0.00
≥3	1,932	4.1	740	4.2		−0.01	726	4.2	730	4.2		0.00
WM hospitalization												
No	17,038	35.7	1,161	6.7	0.151	0.01	6,177	35.9	1,149	6.7	0.157	0.00
Yes	12,468	26.2	3,166	18.1		0.01	4,444	25.8	3,133	18.2		0.00
KM hospitalization												
No	47,678	100	17,217	98.6	<0.001	0.17	17,217	100	17,217	100	–	–
Yes			252	1.4		−0.17						
Spondylolisthesis												
No	46,216	96.9	16,886	96.7	0.079	0.02	16,661	96.8	16,658	96.8	0.083	0.00
Yes	1,462	3.1	583	3.3		−0.02	556	3.2	559	3.3		0.00

### 3.2 Surgical and opioid prescription outcomes in matched cohorts

Kaplan–Meier survival curves for the incidence of lumbar spine surgery and opioid prescriptions in the matched KM and control groups are shown in Supplementary Figures 1–3. Although the KM group exhibited a trend toward lower opioid prescription rates, the difference was not statistically significant based on the log-rank test. Detailed comparisons of opioid prescription rates between the groups are provided in Supplementary Table 5.

Cox proportional hazards regression analyses were conducted with sequential adjustments, including demographic variables (age, sex, insurance type), CCI, and comorbidities.

#### 3.2.1 Surgery

Patients in the KM group had a significantly lower risk of undergoing lumbar spine surgery compared to the control group. In the model adjusted for healthcare utilization, HR was 0.806 (95% CI: 0.768–0.846). In the final model (Model 4), which additionally adjusted for demographic variables, CCI, and comorbidities, the HR was 0.821 (95% CI: 0.782–0.862), indicating that KM utilization within 1 year of diagnosis was significantly associated with a reduced long-term risk of surgery. Sensitivity analyses using higher KM utilization thresholds (≥6 and ≥8 visits during the first year) showed slightly higher hazard ratio estimates than the primary analysis, but the overall direction and interpretation of the association remained unchanged (Supplementary Table 6).

#### 3.2.2 Opioid prescription

For the combined outcome of ≥ 7 days of opioid-class analgesics or ≥ 14 days of tramadol, the KM group showed a significantly lower prescription rate after adjustment for healthcare utilization (HR: 0.794, 95% CI: 0.739–0.854). This association remained significant in the fully adjusted model (HR: 0.810; 95% CI: 0.752–0.872).

For the outcome limited to opioid-class analgesics (excluding tramadol), the KM group again demonstrated a significantly lower prescription rate in the healthcare utilization-adjusted model (HR: 0.729; 95% CI: 0.605–0.877). This association persisted in the final model (HR: 0.761; 95% CI: 0.630–0.919) (Table 2).

**Table 2:** Multivariate Cox regression analysis of surgery and opioid outcomes comparing Korean medicine and Western medicine user groups.

Outcomes	After propensity score matching			
	Model 1^a^	Model 2^b^	Model 3^c^	Model 4^d^
	HR (95% CI)	HR (95% CI)	HR (95% CI)	HR (95% CI)
Surgery (ref. WM)	0.806 (0.768–0.846)^***^	0.805 (0.767–0.845)^***^	0.808 (0.770–0.847)^***^	0.821 (0.782–0.862)^***^
Opioid (ref. WM)	0.794 (0.739–0.854)^***^	0.810 (0.754–0.872)^***^	0.811 (0.754–0.872)^***^	0.810 (0.752–0.872)^***^
Opioids excluding tramadol (ref. WM)	0.729 (0.605–0.877)^***^	0.750 (0.622–0.903)^**^	0.751 (0.623–0.905)^**^	0.761 (0.630–0.919)^**^

a Model 1: Adjusted for healthcare utilization (categorized into quartiles).

b Model 2: Adjusted for healthcare utilization (quartiles) and demographic variables (age, sex, and health insurance type).

c Model 3: Adjusted for healthcare utilization, demographic variables, and CCI.

d Model 4: Adjusted for healthcare utilization, demographic variables, CCI, and comorbidities.

NHI, National Health Insurance; CCI, Charlson Comorbidity Index; HR, hazard ratio; CI, confidence interval. * *p*-value <0.05, ** *p*-value <0.01, *** *p*-value <0.001.

Finally, model fit was evaluated across the various stages of adjustment and matching using the AIC. Results are presented in Supplementary Table 7.

## 4 Discussion

This study is the first to utilize nationwide claims data from HIRA to evaluate the impact of KM on surgery and opioid use among patients with LSS in real-world clinical settings.

Although the log-rank test did not show statistically significant differences between groups, the Kaplan–Meier curves indicated a small but persistent separation over time. When adjusted for demographic and clinical factors in the Cox models, these modest differences translated into statistically significant hazard reductions. This pattern suggests that while the absolute differences were not large enough to reach significance in an unadjusted survival comparison, the adjusted analyses captured clinically meaningful and directionally consistent benefits associated with KM utilization. Given the limitations of claims data in capturing clinical severity, healthcare utilization was used as a proxy for disease severity in our models, and model fit metrics such as the AIC supported the validity of this analytical approach.

The proportion of medical aid beneficiaries in the matched dataset was approximately 5%, slightly higher than the national average of 2.8–2.9%. Although this pattern was observed in both KM and control groups, it may reflect differences in healthcare utilization behaviors compared to the broader LSS population. Therefore, the generalizability of the findings should be interpreted with caution.

Our findings differ slightly from those of previous studies. For example, a nationwide retrospective cohort study by Koh et al. (26), which investigated the impact of acupuncture on surgery rates among patients with low back pain, reported a lower HR of 0.633 (95% CI: 0.576–0.696), compared to the HR of approximately 0.8 observed in our study. This discrepancy may be partly attributable to key methodological and clinical differences between the two studies. Koh et al. examined nonspecific low back pain, a condition with greater heterogeneity and generally less structural degeneration than LSS, and defined acupuncture exposure as only two treatment sessions over 6 weeks, with a two-year follow-up duration. In contrast, our study focused on patients with radiologically inferred degenerative spinal stenosis, applied a more stringent KM exposure definition (≥3 visits over the first year and greater KM than WM utilization), and evaluated long-term outcomes over up to 4 years. These differences in disease severity, exposure intensity, and follow-up duration likely contribute to the more modest effect sizes observed in our LSS cohort.

In Korea, integrative KM is widely used as a conservative and noninvasive treatment for LSS. Clinical practice generally involves a combination of modalities such as acupuncture, herbal medicine, Chuna manual therapy, and pharmacopuncture (13). An analysis of Korean national health insurance data from 2010 to 2019 indicated that the most frequently claimed KM treatments for LSS included acupuncture (50.6%), cupping (12.2%), electroacupuncture (8.7%), and moxibustion (5.7%). Herbal decoctions, pharmacopuncture, and Chuna therapy – although not reimbursed at the time – were also commonly used (27). Notably, Chuna therapy has been reimbursed under insurance since 2019 (27).

The potential for KM to reduce the need for surgery and opioid use is supported by a growing body of clinical and mechanistic evidence. A longitudinal follow-up study by Kim et al. (11) demonstrated that KM treatment, including acupuncture, resulted in sustained improvements in pain and functional disability among patients with LSS. In addition, a retrospective observational study reported that integrative KM treatment was associated with meaningful improvements in gait function and pain over a one-year period, supporting its potential long-term benefits in routine practice (12).

Mechanistically, acupuncture has been shown to stimulate the posterior rami of the spinal nerves, enhance blood flow to the sciatic and cauda equina nerves, and modulate inflammatory pathways by inhibiting HMGB1/RAGE and TLR4/NF-κB signaling, thereby reducing pro-inflammatory mediators such as IL-1β, IL-6, and PGE2 (28, 29). Herbal medicines commonly used in KM practice for LSS, such as *Harpagophytum procumbens,* has demonstrated anti-inflammatory and antioxidant effects through NRF2 activation, while human placental extract pharmacopuncture has been shown to downregulate pain-related gene expression and promote neuroregeneration (30, 31).

Building on these mechanisms, recent randomized evidence further supports the clinical relevance of KM interventions. A pragmatic randomized pilot trial of acupotomy showed improvements in pain, walking capacity, and disability over 12 weeks with no major safety concerns (14). Moreover, a multicenter pragmatic randomized controlled trial investigating pharmacopuncture for LSS is currently underway (32), and its findings are expected to provide additional high-quality evidence on the effectiveness of KM modalities in managing degenerative lumbar conditions.

Despite the strengths of this large, population-based study, several limitations should be noted. First, the reliance on administrative claims data precludes access to key clinical variables such as anatomical stenosis severity, symptom duration, neurogenic claudication, functional status, and patient-reported pain outcomes. The absence of these details may introduce residual confounding and limits our ability to establish clinical equivalence between groups. Second, although propensity score matching was used to balance observable characteristics, treatment selection bias may remain, as patients who choose KM may have stronger preferences for nonsurgical or nonpharmacologic care. To partially account for socioeconomic differences, insurance type was used as a proxy indicator of socioeconomic status; however, direct measures such as income or education are unavailable in claims data and may contribute to unmeasured confounding.

Third, our analysis included only reimbursed KM and WM services. Non-reimbursed treatments—such as herbal decoctions, pharmacopuncture, certain WM procedures, and over-the-counter medications—were not captured, potentially underestimating the full range of treatments received by patients. Fourth, although the washout period was selected to identify newly diagnosed patients, LSS is a chronic and recurrent condition, and a one-year washout may not exclude all prior disease activity.

Finally, while subgroup analyses could offer insight into heterogeneity of treatment effects by age, comorbidity burden, or other factors, we elected not to perform extensive subgroup analyses to avoid multiplicity and overinterpretation in this large administrative dataset. Future studies with richer clinical information and predefined subgroup criteria will be needed to clarify which patient groups benefit most from KM interventions.

From a clinical perspective, these findings also suggest ways in which KM could be incorporated into integrative care pathways for LSS. KM modalities such as acupuncture, Chuna manual therapy, and pharmacopuncture may be positioned as early conservative options before considering opioid therapy or surgical referral, particularly for patients seeking nonpharmacologic management. Integrative medicine approaches—such as shared decision-making between KM and WM providers, cross-disciplinary referrals, and combined rehabilitation programs—may further enhance symptom control while reducing reliance on invasive or opioid-based treatments. Developing such integrative pathways can help translate KM’s real-world benefits into routine clinical practice.

Nonetheless, our findings suggest that KM may offer a meaningful nonsurgical and nonpharmacological treatment option for patients with LSS, particularly in aging populations. Future research should aim to confirm these findings through randomized controlled trials involving patients with comparable clinical characteristics and stricter inclusion criteria.

In conclusion, this nationwide retrospective cohort study used HIRA real-world data to assess the impact of KM treatment on surgery and opioid prescription rates among patients with LSS. The results showed that patients who received KM care had significantly lower long-term rates of both surgery and opioid use compared to those who did not. These findings provide real-world evidence that KM may serve as an effective conservative treatment strategy for LSS, potentially reducing reliance on surgical and pharmacologic interventions. These insights may support informed decision-making by clinicians, patients, and policymakers. However, further large-scale, prospective randomized controlled trials are needed to validate these findings and strengthen the evidence base for KM in LSS management.

## Data Availability

The datasets presented in this article are not readily available because the data used in this study come from the Health Insurance Review and Assessment Service (HIRA) in Korea. To protect patient privacy, access to the data is limited to certified researchers within South Korea, with analysis permitted only within the HIRA system and data export strictly prohibited. Requests to access the datasets should be directed to YL, goodsmile8119@gmail.com.
