# Supplementary material for: Effect of Korean medicine treatment on surgery and opioid prescription among patients with lumbar spinal stenosis: a nationwide retrospective cohort study
Source: Front Med (Lausanne). 2026 Jan 16;13:1703911. doi: 10.3389/fmed.2026.1703911 (PMC12855126; doi:10.3389/fmed.2026.1703911)
Supplement: Supplementary file 1 [file Data_Sheet_1.docx]

| **CODE** | **Full_name** |
| --- | --- |
| C00-C97 | Malignant neoplasms |
| V01-V99 | Transport accidents |
| D16.6 | Benign neoplasm of Vertebral column |
| D32.1 | Benign neoplasm of Spinal meninges |
| D33.4 | Benign neoplasm of Spinal cord |
| M45 | Ankylosing spondylitis |
| M86 | Osteomyelitis |
| S32 | Fracture of lumbar spine and pelvis |
| G06.1 | Intraspinal abscess and granuloma |
| M46.0 | Spinal enthesopathy |
| M46.9 | Inflammatory spondylopathy, unspecified |
| M89.6 | Osteopathy after poliomyelitis |
| M90.2 | Osteopathy in other infectious diseases classified elsewhere |
| T09.3 | Injury of spinal cord, level unspecified |
| T08.1 | Fracture of spine, level unspecified, open |
| S34.1 | Other injury of lumbar spinal cord |
| S34.3 | Injury of cauda equina |

Supplementary Table 1. Red flags

Supplementary Table 2. Reimbursement Codes and Procedure Names for Lumbar Spine Surgeries

| **CODE** | **Full_name** |
| --- | --- |
| N1493 | Diskectomy(Invasive)-Lumbar Spine |
| N1494 | Diskectomy By Endoscopy |
| N1499 | Laminectomy, Lumbar Spine |
| N2499 | Laminectomy, Lumbar Spine |
| N0466 | Arthrodesis of Spine-Lumbar Spine-Anterior Technique |
| N1466 | Arthrodesis of Spine-Lumbar Spine-Anterior Technique |
| N0469 | Arthrodesis of Spine-Lumbar Spine-Posterior Technique |
| N1469 | Arthrodesis of Spine-Lumbar Spine-Posterior Technique |
| N2470' | Posterior Lumbar Interbody Fusion |
| N1460 | Posterior Lumbar Interbody Fusion |

Supplementary Table 3. ATC Codes Used for the Analysis of Opioid Analgesics

| Drug | ATC_code | Full_name |
| --- | --- | --- |
| Tramadol | N02AJ13 | tramadol and paracetamol |
|  | N02AX02 | tramadol |
|  | N02AX52 | tramadol, combinations |
| Opioid | N02AA01 | morphine |
|  | N02AA03 | hydromorphone |
|  | N02AA05 | oxycodone |
|  | N02AA08 | dihydrocodeine |
|  | N02AA55 | oxycodone and naloxone |
|  | N02AB02 | pethidine |
|  | N02AB03 | fentanyl |
|  | N02AE01 | buprenorphine |
|  | N02AF01 | butorphanol |
|  | N02AF02 | nalbuphine |
|  | N02AJ | Opioids in combination with non-opioid analgesics |
|  | N02AJ09 | codeine and other non-opioid analgesics |
|  | N02AX06 | tapentadol |

Supplementary Table 4. Baseline characteristics by covariates not used in propensity score matching

| Variables | Before propensity score matching | | | | | After propensity score matching | | | | |
| --- | --- | --- | --- | --- | --- | --- | --- | --- | --- | --- |
|  | WM | | KM | | p-value | WM | | KM | | p-value |
|  | N | % | N | % |  | N | % | N | % |  |
| **Surgery dataset** | 245,804 |  | 72,005 |  |  | 70,897 |  | 70,897 |  |  |
| **Comorbidity** |  |  |  |  |  |  |  |  |  |  |
| Disorders of nerve roots and spinal cord | 2,224 | 0.9 | 708 | 1.0 | 0.053 | 622 | 0.9 | 688 | 1.0 | 0.067 |
| Spondylolisthesis | 8,313 | 3.4 | 2,631 | 3.7 | <0.001 | 2,545 | 3.6 | 2,546 | 3.6 | 0.317 |
| Scoliosis | 1,689 | 0.7 | 586 | 0.8 | <0.001 | 504 | 0.7 | 572 | 0.8 | 0.037 |
| Other arthrosis | 107,673 | 43.8 | 33,206 | 46.1 | <0.001 | 32,779 | 46.2 | 32,785 | 46.1 | 0.973 |
| Rheumatoid arthritis | 7,326 | 3.0 | 2,275 | 3.2 | 0.014 | 2,223 | 3.1 | 2,237 | 3.2 | 0.830 |
| Osteoporosis | 32,601 | 13.3 | 11,368 | 15.8 | <0.001 | 10,920 | 15.4 | 11,241 | 15.9 | 0.013 |
| Mental disorder | 52,051 | 21.2 | 16,132 | 22.4 | <0.001 | 15,526 | 21.9 | 15,913 | 22.5 | 0.011 |
| Other deforming dorsopathies | 1,902 | 0.8 | 601 | 0.8 | 0.104 | 557 | 0.8 | 592 | 0.8 | 0.301 |
| Spondylosis | 44,786 | 18.2 | 13,336 | 18.5 | 0.066 | 13,389 | 18.9 | 13,130 | 18.5 | 0.075 |
| Other spondylopathies | 46,789 | 19.0 | 13,141 | 18.3 | <0.001 | 13,790 | 19.5 | 12,912 | 18.2 | <0.001 |
| Dorsalgia | 143,031 | 58.2 | 53,290 | 74.0 | <0.001 | 41,878 | 59.1 | 52,435 | 74.0 | <0.001 |
| Biomechanical lesions | 3,136 | 1.3 | 1,184 | 1.6 | <0.001 | 928 | 1.3 | 1,157 | 1.6 | <0.001 |
| Dislocation | 49,663 | 20.2 | 20,553 | 28.5 | <0.001 | 14,039 | 19.8 | 20,110 | 28.4 | <0.001 |
| **Healthcare Utilization** |  |  |  |  | <0.001 |  |  |  |  | <0.001 |
| Q1 (0-25%) | 87,406 | 35.6 | 6,924 | 9.6 |  | 25,067 | 35.4 | 6,816 | 9.6 |  |
| Q2 (26-50%) | 59,263 | 24.1 | 11,884 | 16.5 |  | 17,074 | 24.1 | 11,718 | 16.5 |  |
| Q3 (51-75%) | 54,930 | 22.4 | 19,195 | 26.7 |  | 15,858 | 22.4 | 18,956 | 26.7 |  |
| Q4 (76-100%) | 44,205 | 18.0 | 34,002 | 47.2 |  | 12,898 | 18.2 | 12,898 | 47.1 |  |
| **Opioid dataset** | 47,678 |  | 17,469 |  |  | 17,217 |  | 17,217 |  |  |
| **Comorbidity** |  |  |  |  |  |  |  |  |  |  |
| Disorders of nerve roots and spinal cord | 333 | 0.7 | 139 | 0.8 | 0.195 | 94 | 0.6 | 135 | 0.8 | 0.007 |
| Spondylolisthesis | 1,462 | 3.1 | 583 | 3.3 | 0.079 | 556 | 3.2 | 559 | 3.3 | 0.083 |
| Scoliosis | 304 | 0.6 | 125 | 0.7 | 0.276 | 1.2 | 0.6 | 121 | 0.7 | 0.201 |
| Other arthrosis | 15,426 | 32.4 | 6,106 | 35.0 | <0.001 | 5,960 | 34.6 | 6,032 | 35.0 | 0.398 |
| Rheumatoid arthritis | 983 | 2.1 | 387 | 2.2 | 0.226 | 395 | 2.3 | 383 | 2.2 | 0.660 |
| Osteoporosis | 4,975 | 10.4 | 2,159 | 12.4 | <0.001 | 2,110 | 12.3 | 2,130 | 12.4 | 0.731 |
| Mental disorder | 7,614 | 16.0 | 2,933 | 16.8 | 0.012 | 2,862 | 16.6 | 2,892 | 16.8 | 0.657 |
| Other deforming dorsopathies | 306 | 0.6 | 107 | 0.6 | 0.677 | 105 | 0.6 | 106 | 0.6 | 0.945 |
| Spondylosis | 6,305 | 13.2 | 2,428 | 13.9 | 0.025 | 2,368 | 13.8 | 2,393 | 13.9 | 0.696 |
| Other spondylopathies | 7,522 | 15.8 | 2,541 | 14.6 | <0.001 | 2,792 | 16.2 | 2,506 | 14.6 | <0.001 |
| Dorsalgia | 22,873 | 48.0 | 11,607 | 66.4 | <0.001 | 8,381 | 48.7 | 11,431 | 66.4 | <0.001 |
| Biomechanical lesions | 476 | 1.0 | 265 | 1.5 | <0.001 | 158 | 0..9 | 253 | 1.5 | <0.001 |
| Dislocation | 8,685 | 18.2 | 4,559 | 26.1 | <0.001 | 3,048 | 17.7 | 4,456 | 25.9 | <0.001 |
| **Healthcare Utilization** |  |  |  |  | <0.001 |  |  |  |  | <0.001 |
| Q1 (0-25%) | 17,038 | 35.7 | 1,161 | 6.7 |  | 6,177 | 35.9 | 1,149 | 6.7 |  |
| Q2 (26-50%) | 12,468 | 26.2 | 3,166 | 18.1 |  | 4,444 | 25.8 | 3,133 | 18.2 |  |
| Q3 (51-75%) | 10,647 | 22.3 | 4,727 | 27.1 |  | 3,846 | 22.3 | 4,670 | 27.1 |  |
| Q4 (76-100%) | 7,525 | 15.8 | 8,415 | 48.2 |  | 2,750 | 16.0 | 8,265 | 48.0 |  |

**Supplementary Table 5. Opioid Use in Korean medicine and Western medicine user groups**

|  |  |  | WM | | | | KM | | | |
| --- | --- | --- | --- | --- | --- | --- | --- | --- | --- | --- |
| Drug | ATC_code | full_name | Before Propensity Score Matching | | After Propensity Score Matching | | Before Propensity Score Matching | | After Propensity Score Matching | |
|  |  |  | Claim | Patient | Claim | Patient | Claim | Patient | Claim | Patient |
| Tramadol | N02AJ13 | tramadol and paracetamol | 144,471 (50.5) | 24,926 (46.7) | 53,123 (50.2) | 9,127 (46.7) | 50,579 (48.6) | 9,251 (46.1) | 50,006 (48.6) | 9,136 (46.1) |
|  | N02AX02 | tramadol | 141,631 (49.5) | 28,413 (53.3) | 52,771 (49.8) | 10,428 (53.3) | 53,390 (51.4) | 10,830 (53.9) | 52,788 (51.4) | 10,675 (53.9) |
| Opioid | N02AA01 | morphine | 1,135 (6.0) | 900 (7.3) | 430 (6.5) | 353 (7.8) | 408 (6.2) | 319 (7.0) | 404 (6.2) | 315 (7.1) |
|  | N02AA03 | Hydromorphone | 184 (1.0) | 131 (1.1) | 73 (1.1) | 57 (1.3) | 71 (1.1) | 50 (1.1) | 70 (1.1) | 49 (1.1) |
|  | N02AA05 | oxycodone | 396 (2.1) | 238 (1.9) | 135 (2.0) | 87 (1.9) | 106 (1.6) | 69 (1.5) | 102 (1.6) | 68 (1.5) |
|  | N02AA08 | Dihydrocodeine | 22 (0.1) | 17 (0.1) | 7 (0.1) | 5 (0.1) | 6 (0.1) | 5 (0.1) | 6 (0.1) | 5 (0.1) |
|  | N02AA55 | oxycodone and naloxone | 441 (2.3) | 255 (2.1) | 139 (2.1) | 80 (1.8) | 166 (2.5) | 99 (2.2) | 163 (2.5) | 97 (2.2) |
|  | N02AB02 | pethidine | 9,773 (51.9) | 7,275 (58.7) | 3,415 (51.3) | 2,583 (57.4) | 3,545 (53.6) | 2,731 (60.3) | 3,492 (53.5) | 2,693 (60.3) |
|  | N02AB03 | fentanyl | 808 (4.3) | 488 (3.9) | 293 (4.4) | 177 (3.9) | 292 (4.4) | 199 (4.4) | 292 (4.5) | 199 (4.5) |
|  | N02AE01 | buprenorphine | 4,021 (21.3) | 1,823 (14.7) | 1,445 (21.7) | 697 (15.5) | 1,339 (20.3) | 650 (14.4) | 1,326 (20.3) | 640 (14.3) |
|  | N02AF01 | butorphanol | 92 (0.5) | 78 (0.6) | 38 (0.6) | 31 (0.7) | 38 (0.6) | 33 (0.7) | 37 (0.6) | 32 (0.7) |
|  | N02AF02 | nalbuphine | 153 (0.8) | 136 (1.1) | 59 (0.9) | 53 (1.2) | 35 (0.5) | 34 (0.8) | 35 (0.5) | 34 (0.8) |
|  | N02AJ | Opioids in combination with non-opioid analgesics | 110 (0.6) | 78 (0.6) | 47 (0.7) | 27 (0.6) | 26 (0.4) | 20 (0.4) | 25 (0.4) | 19 (0.4) |
|  | N02AJ09 | codeine and other non-opioid analgesics | 1,513 (8.0) | 844 (6.8) | 491 (7.4) | 293 (6.5) | 495 (7.5) | 269 (5.9) | 491 (7.5) | 266 (6.0) |
|  | N02AX06 | tapentadol | 195 (1.0) | 129 (1.0) | 90 (1.4) | 57 (1.3) | 81 (1.2) | 50 (1.1) | 81 (1.2) | 50 (1.1) |

**Supplementary Table 6. Sensitivity analyses of surgery outcomes using alternative thresholds for Korean medicine utilization**

|  | After Propensity Score Matching |  |
| --- | --- | --- |
|  | HR (95% CI) | |
| Surgery (≥6 visits)  (ref. WM) | 0.896  (0.855-0.938) ^***^ | |
| Surgery (≥8 visits)  (ref. WM) | 0.892  (0.847-0.940) *** | |

**Supplementary Table 7. Cox AIC (Akaike Information Criterion)**

| Surgery |  | Before Propensity Score Matching | | | | After Propensity Score Matching | | | |
| --- | --- | --- | --- | --- | --- | --- | --- | --- | --- |
|  |  | Model1 | Model2 | Model3 | Model4 | Model1 | Model2 | Model3 | Model4 |
|  | AIC | 433330 | 431657 | 431564 | 430878 | 181006 | 180332 | 180275 | 180004 |
| Opioid |  | Before Propensity Score Matching | | | | After Propensity Score Matching | | | |
|  |  | Model1 | Model2 | Model3 | Model4 | Model1 | Model2 | Model3 | Model4 |
|  | AIC | 144427 | 144043 | 144019 | 143772 | 77120 | 71930 | 71920 | 71798 |
| Opioid  (excluding tramadol) |  | Before Propensity Score Matching | | | | After Propensity Score Matching | | | |
|  |  | Model1 | Model2 | Model3 | Model4 | Model1 | Model2 | Model3 | Model4 |
|  | AIC | 22669 | 22564 | 22558 | 22535 | 11153 | 11104 | 11103 | 11110 |
